# Treatment with mononuclear cell populations improves post-infarction cardiac function but does not reduce arrhythmia susceptibility

**DOI:** 10.1371/journal.pone.0208301

**Published:** 2019-02-14

**Authors:** René P. Andrié, Thomas Beiert, Vincent Knappe, Markus Linhart, Florian Stöckigt, Alexandra M. Klein, Alexander Ghanem, Indra Lübkemeier, Wilhelm Röll, Georg Nickenig, Bernd K. Fleischmann, Jan W. Schrickel

**Affiliations:** 1 Department of Cardiology, University of Bonn, Bonn, Germany; 2 Institute of Physiology I, Life & Brain Center, University of Bonn, Bonn, Germany; 3 Department of Cardiology, Asklepios Hospital Hamburg, Hamburg, Germany; 4 LIMES-Institute, Molecular Genetics, University of Bonn, Bonn, Germany; 5 Department of Cardiovascular Surgery, University of Bonn, Bonn, Germany; University of Minnesota, UNITED STATES

## Abstract

**Background:**

Clinical and experimental data give evidence that transplantation of stem and progenitor cells in myocardial infarction could be beneficial, although the underlying mechanism has remained elusive. Ventricular tachyarrhythmia is the most frequent and potentially lethal complication of myocardial infarction, but the impact of mono nuclear cells on the incidence of ventricular arrhythmia is still not clear.

**Objective:**

We aimed to characterize the influence of splenic mononuclear cell populations on ventricular arrhythmia after myocardial infarction.

**Methods:**

We assessed electrical vulnerability *in vivo* in mice with left ventricular cryoinfarction 14 days after injury and intramyocardial injection of specific subpopulations of mononuclear cells (MNCs) (CD11b-positive cells, Sca-1-positive cells, early endothelial progenitor cells (eEPCs)). As positive control group we used embryonic cardiomyocytes (eCMs). Epicardial mapping was performed for analysing conduction velocities in the border zone. Left ventricular function was quantified by echocardiography and left heart catheterization.

**Results:**

*In vivo* pacing protocols induced ventricular tachycardia (VT) in 30% of non-infarcted mice. In contrast, monomorphic or polymorphic VT could be evoked in 94% of infarcted and vehicle-injected mice (p<0.01). Only transplantation of eCMs prevented post-infarction VT and improved conduction velocities in the border zone in accordance to increased expression of connexin 43. Cryoinfarction resulted in a broad aggravation of left ventricular function. All transplanted cell types augmented left ventricular function to a similar extent.

**Conclusions:**

Transplantation of different MNC populations after myocardial infarction improves left ventricular function similar to effects of eCMs. Prevention of inducible ventricular arrhythmia is only seen after transplantation of eCMs.

## Introduction

Ischemic cardiomyopathy is a leading cause of morbidity and mortality. Transmural myocardial infarction (MI) is characterized by an irreversible loss of cardiomyocytes and formation of scar tissue resulting in heart failure symptoms due to an impairment of left ventricular function. Moreover, scar tissue is a central risk factor for ventricular tachycardia (VT) and sudden cardiac death because it constitutes anatomical conduction barriers that promote electrical re-entrant circuits [1]. Secondary to this structural remodelling, an electrical remodelling with down-regulation of connexin 43 (Cx43) occurs [2]. Therefore, new therapeutic approaches for treatment of heart failure should be focused on (a) improvement of left ventricular function in addition to reperfusion therapy in acute MI and (b) reduction of ventricular arrhythmia. Cell transplantation has emerged as a potential treatment strategy for heart failure secondary to MI. Different autologous cell types, in particular bone marrow (BM) derived stem and progenitor cells, are used in clinical trials in patients after MI. Studies showed conflicting results concerning the improvement of left ventricular function [3]. The 5-year follow-up of the TOPCARE-AMI trial showed a persistence of the beneficial effects on left ventricular function during long-term follow-up [4]. Recently, a meta-analysis could demonstrate that intracoronary infusion of bone marrow cells was associated with a moderate improvement of left ventricular function [5]. Meanwhile, so called paracrine/humoral effects have been proposed as main mechanisms for the therapeutic effects of stem and progenitor cells. Additionally, several studies could convincingly demonstrate that transdifferentiation of BM cells into neither cardiomyocytes nor endothelial cells occurs [6]. In addition, neither BM derived cells nor skeletal myoblasts (SMs) couple electrically with the host myocardium in a significant manner [7,8]. Notably, after transplantation of SMs VTs have been reported in several patients [9]. Therefore, cellular cardiomyoplasty may offer pro-arrhythmic potential in some cell types. In a mouse model of MI transplantation of embryonic cardiomyocytes (eCMs) resulted in an effective engraftment with expression of gap junction proteins, augmentation of left ventricular function and strongly reduced inducibility of VT [10]. Genetic modification of SMs expressing Cx43 also eliminated the pro-arrhythmic effects [10] indicating that electrical coupling with the host myocardium is a prerequisite for anti-arrhythmic effects of grafted cells. In line with these findings, Shiba et al. found no arrhythmic potential in animals treated with human embryonic stem cell-derived cardiomyocytes (hESC-CMs) [11,12]. On the other hand, despite their benefits, also pro-arrhythmic effects were described in an animal model with monkeys [13] and also mice [14].

Data concerning pro- or anti-arrhythmic effects of BM derived stem and progenitor cells after transplantation are rare despite its wide propagation in clinical trials.

The spleen represents a large source of undifferentiated monocytes that closely resemble blood monocytes, but not BM derived monocytes [15]. In ischemia-reperfusion injury, splenectomy significantly reduces MI size since inflammation maintains the deterioration of cardiac function [15,16], but in contrast unreperfused MI requires clearance of a large amount of necrotic cardiomyocytes and replacement by fibrotic scar tissue. Proper infarct healing critically depends on inflammatory (phase 1) and reparative (phase 2) actions of monocytes, and abrogation/prolongation of phase 1 as well as abrogation of phase 2 would be deleterious, such as insufficient presence of monocytes is associated with impaired infarct healing [15]. For example, Leuschner et al. could demonstrate accelerated LV remodeling and reduced LV function after MI in splenectomized mice [17].

Both spleen derived mononuclear cells (MNCs) and subsequently *in vitro* differentiated endothelial progenitor cells (EPCs) have been shown to improve vascular repair [18,19] with potentially higher effectiveness of MNCs [18]. Furthermore, transplantation of EPCs was demonstrated to improve left ventricular function [20], but the impact on electrical stability is unknown. Therefore we aimed to to systematically evaluate the effects of cell transplantation of splenic MNCs and EPCs on ventricular vulnerability in comparison to eCMs in the setting of acute myocardial infarction in mice *in vivo*.

Since MNCs comprise of various cell types we additionally investigated the stem cell antigen-1 (Sca-1) and CD11b positive MNC subpopulations. Sca-1 was initially introduced as a marker of hematopoietic stem cells, but later also Sca-1 positive somatic stem cells were described with potential for heart regeneration [21]. CD11b, being expressed on monocytes, macrophages, granulocytes and natural killer cells, is involved in adhesion and migration of these cells to sites of inflammation.

## Material and methods

All animal experiments were performed in accordance with National Institutes of Health animal protection guidelines and were approved by the local authorities.

### Preparation of MNCs, CD11b-positive and stem cell antigen-1 (Sca-1)-positive cells

Ubiquitous red fluorescent mice (Ds-Red.T3, CD1-background) were sacrificed by cervical dislocation, spleens were explanted and mechanically minced. MNCs were isolated using a Ficoll gradient (Lympholite-M, Cedarlane). For preparation of CD11b- and Sca-1-positive cells, the same amount of spleen-derived MNCs was subjected to magnetic bead separation. In brief, spleen-derived MNCs were washed, re-suspended and mixed with colloidal superparamagnetic microbeads conjugated to CD11b or Sca-1 antibodies (MACS MicroBeads, Miltenyi Biotec). After incubation and additional washing, magnetic cell separation was performed by filling the cell suspension into a depletion column placed in the magnetic field of a magnetic bead separator (MACS Depletion Columns; MidiMACS Separator, Miltenyi). The collected effluent contained the negative MNC fraction depleted of CD11b- or Sca-1-positive cells, respectively. After taking the column out of the magnetic field, the attached CD11b-/Sca-1-positive MNCs were collected in buffer.

### Preparation of spleen-derived endothelial progenitor cells (eEPCs)

Spleen-derived MNCs from Ds-Red.T3-mice were seeded on fibronectin-coated 24-well plates in 0.5 mL of endothelial basal medium (EBM) (CellSystems) supplemented with 1 μg/mL hydrocortisone, 3 μg/mL bovine brain extract, 30 μg/mL gentamicin, 50 μg/mL amphotericin B, 10 μg/mL human endothelial growth factor (hEGF) and 20% fetal calf serum (FCS). After 4 days in culture, cells were extensively washed with normal saline and adherent cells were counted and resuspended in normal saline solution.

### Preparation of embryonic cardiomyocytes (eCMs)

Embryonic ventricular cardiomyocytes (E13.5–E16.5) of transgenic mice (HIM:OF1-strain) expressing enhanced green fluorescent protein (eGFP) under a cardiac specific promoter were harvested as reported earlier [10]. For intramyocardial injection the cells were re-suspended in DMEM containing 10% FCS (20,000 cells/μL).

### Cryoinfarction and cell transplantation

Recipient mice (12 weeks old CD1 mice) were anesthetized, intubated and mechanically ventilated as described before [10]. Reproducible transmural cryolesions at the anterior-lateral left ventricular wall were generated with a liquid nitrogen-cooled copper probe (diameter 3.5 mm) and cells suspended in 5–6 μl solution were injected intramyocardially immediately thereafter (500,000 MNCs, 500,000 CD11b-positive cells, 500,000 Sca-1-positive cells, 500,000 eEPCs, 500,000 eCMs; n = 15–20 animals per group). In control mice the same amount of medium without cells was injected.

### Echocardiographic studies

Echocardiography was performed 14 days after MI and cellular replacement therapy by 1D- and 2D-echo by two blinded investigators. Studies were performed at rest and under inotropic stimulation with intravenous dobutamine at low-dose (10 μg/kg/min). High-resolution echocardiography was performed using a commercially available ultrasound system (HDI-5000, Philips Medical Systems, Netherlands), equipped with a 15 MHz linear array transducer in harmonic imaging mode. Mice were anesthetized with isoflurane (2 Vol.-% for induction and 0.8–1.2 Vol.-% isoflurane in O_2_ for maintenance). Heart rate was monitored continuously by use of a 6-lead surface electrocardiogram (ECG) to recognize and counteract cardiodepressant effects (AD-Instruments, Castle Hill, Australia). Chest fur was carefully depilated and a layer of centrifuged contact gel served as coupling medium allowing data acquisition without thoracic compression. 1D- and 2D-echo were performed as described previously [22]. Briefly, 2D-guided M-mode data was acquired in the parasternal short-axis view at the level of the papillary muscle. For 2D-echo, two positions of the short-axis scan plane were selected and compared: a) at the mid-papillary imaging plane (3.5 mm apical of the aortic valve level) and b) at the basis of the papillary muscles (4.5 mm apical of the aortic valve level). Fractional shortening (FS) of the left ventricle (LV) was calculated as follows: FS = [(LVEDD—LVESD)/LVEDD] x 100.

### Left heart catheterization

Left ventricular catheterization was performed two weeks after MI by a blinded investigator with a 1.4 French Millar Aria1 catheter (ARIA SPR-719, Millar Instruments Inc, Houston, TX). Mice were anesthetized by isoflurane (1.0 Vol.-% isoflurane in O_2_ for maintenance), intubated and ventilated. The catheter was positioned in the LV via the right carotid artery for continuous registration of LV pressure-volume loops. Systolic function and myocardial contractility were quantified by LV end-systolic pressure, peak rate of rise in LV pressure (dP/dt_max_), ejection fraction (EF), cardiac output, end-systolic volume and stroke volume. Diastolic performance was measured by LV end-diastolic pressure, peak dP/dt_min_ and end-diastolic volume.

### Surface ECG and in vivo transvenous electrophysiological investigation

Mice were put under inhalation anaesthesia and a surface 6-lead ECG was obtained. R-R interval, P-wave duration, PQ interval, QRS duration and QT interval were measured by successive evaluation as described previously [10,23,24]. After amplification, all data were sampled at 4 kHz (Bard stamp amplifier, C.R. Bard Inc.). For transvenous electrophysiological investigation a 2 French octapolar mouse-electrophysiological catheter (CIBer Mouse, NuMed Inc.) was inserted into the right jugular vein and positioned in the right atrium and ventricle. Bipolar electrograms were obtained from adjacent electrode pairs. Double pacing threshold rectangular stimulus pulses were administered by a multi-programmable stimulator (Model 5328, Medtronic). Evaluation of electrophysiological parameters included sinus node recovery period (SNRP), Wenckebach periodicity (WBP) as well as atrial refractory period (ARP), atrio-ventricular-nodal refractory period (AVNRP) and ventricular refractory period (VRP). Vulnerability to VT, characterized by atrio-ventricular dissociation, was tested by ventricular burst stimulations performed at a stimulation cycle length (S1S1) starting at 50 ms with 10-ms stepwise reduction down to 10 ms. In addition, extrastimulus pacing was used with seven fixed-rate stimuli at S1S1 of 120, 110 and 100 ms, followed by up to three short-coupled extra beats with successive reduction of the coupling interval until VRP was reached. Stimulations were performed at twice pacing threshold. VT was defined as at least four ventricular beats. Analyses were performed by a blinded investigator.

### Epicardial mapping of Langendorff-perfused hearts

We determined spontaneous conduction velocities of Langendorff-perfused hearts by epicardial activation mapping with a 128-electrode array as recently described [23,24]. For recording of the infarct border zone electrodes were positioned in part on top of the macroscopically visible scar. Accordingly, the remaining electrodes were spanning the border zone yielding distinct electrograms. Therefore, the scar tissue is approximately located in the area of slowest conduction in the respective epicardial electro-anatomical maps.

### Immunohistochemistry and immunofluorescence

After electrophysiological investigation and left heart catheterization mice were sacrificed by cervical dislocation. Hearts were harvested, imaged with a fluorescence stereomicroscope (Leica MZ 16F, Leica Microsystems) and a ProgRes C101 camera (Jenoptik), arrested in diastole by coronary perfusion with cardioplegic solution at stable haemodynamic pressure and fixed with 4% paraformaldehyde by perfusion. The hearts were cryopreserved and cut into tissue slices 8 or 20 μm thick (for morphometry and immunohistochemistry) and 20 μm thick (for histology). The extent of myocardial lesions in the different groups of animals was determined by Sirius red staining (Aldrich Chemical Company). The area of engraftment (dsRed- or GFP-positive) was measured using ImageJ software (Ver. 1.51s) in macroscopic images of the murine heart and is expressed in relation to the infarcted area.

### Analyses of Cx43 expression

Unfixed frozen hearts for immunohistochemical stainings were embedded in TissueTek OCT medium (Sakura Finetek, Germany) and processed to 10 μm cryosections. After fixing with 4% PFA in PBS, sections were washed three times with 0.1% Tween in TBS and blocked with 5% BSA, 0.1% Tween in TBS for one hour at room temperature. Sections were then incubated with rabbit anti-Cx43 serum (1:1000) diluted in blocking solution at 4°C overnight. On the next day, the sections were washed three times with 0.1% Tween in TBS and incubated with secondary donkey anti-rabbit antibody conjugated with Alexa 488 (1:1000, Molecular Probes, Invitrogen, USA) diluted in blocking solution for one hour at room temperature. After washing the sections twice with 0.1% Tween in TBS, nuclei were stained by incubating sections in 0.1% Tween in TBS with 0.5 mg/ml bisbenzimide (1:1000, Hoechst 33258 stain; Sigma, Germany) for 10 minutes at room temperature. Sections were mounted with Dako Glycergel mounting medium (Dako North America, USA) and viewed with a confocal Laser Scanning Microscope (LSM 710 DuoScan, Zeiss, Germany).

### Statistical analysis

Statistical analysis was performed with a multivariate one-way analysis of variance with post hoc subgroup testing when appropriate (Tukey–Kramer multiple comparisons test) or by Student’s t-test. Non-parametric variables were evaluated by using Kruskal–Wallis testing with Dunn post hoc testing when appropriate. Discrete variables were analysed by two-sided Fisher’s exact test. P<0.05 was regarded as statistically significant. Unless stated otherwise, errors are given as standard deviation (SD).

## Results

### Impact of cellular replacement therapy on ventricular vulnerability

Cryoinfarction resulted in a significant prolongation of QRS duration and QT interval compared to non-infarcted animals (p<0.05; data not shown). R-R interval, P-wave duration and PQ interval were unchanged. There were no significant differences between the MI group and the MNC cell treatment groups in any ECG criteria. The eCM group showed a significant reduction of QRS duration and QT interval. Invasive quantification of SNRP, WBP, ARP and AVNRP was without differences between the groups (data not shown). VRP was significantly decreased in infarcted mice compared to non-infarcted mice (27.2 ms versus 31.6 ms; p<0.05). Transplantation of eCMs resulted in an increase of VRP compared to the MI group (30.9 ms; p<0.05), whereas the MNC groups showed no significant effects.

**Fig 1:**
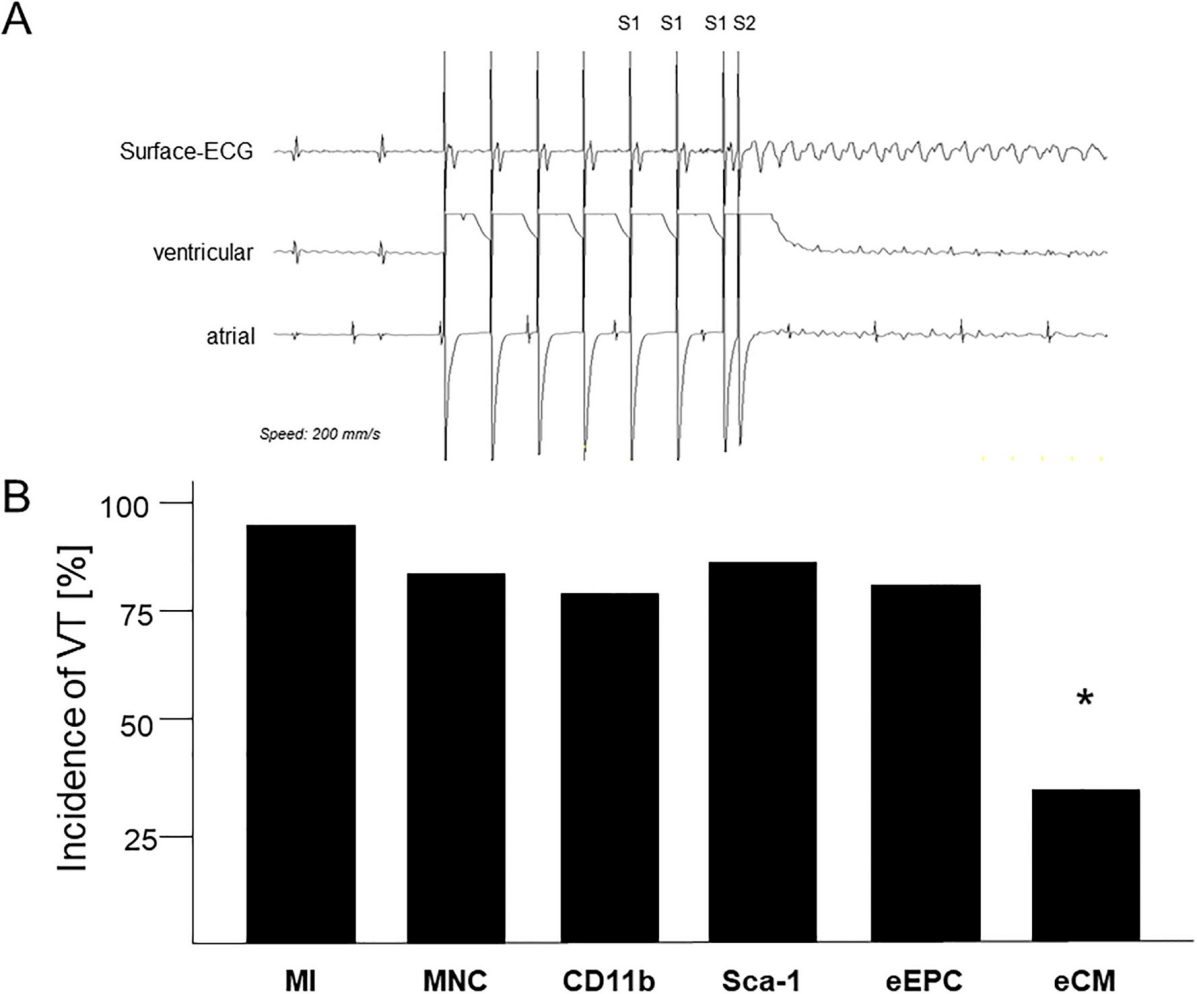
Susceptibility to ventricular tachycardia after myocardial infraction. (A) Induction of ventricular tachycardia (VT) by programmed stimulation in a mouse with myocardial infarction (MI). Note the atrio-ventricular dissociation observed in atrial and ventricular electrograms. (B) Results of *in vivo* electrophysiological examinations with incidence of VT. n = 15–20 per group. * p<0.05 vs. MI.

Vulnerability to VT, characterized by atrio-ventricular dissociation, was tested *in vivo* by ventricular stimulation protocols including programmed and burst stimulation (Fig 1A). VT was induced in only 30% of non-infarcted control mice. In contrast, monomorphic or polymorphic VT could be evoked in 94% of infarcted and vehicle-injected mice (p<0.01) (Fig 1B). All MNC groups showed no difference in VT incidence compared to animals without cell therapy. In clear contrast, the eCM group showed a significant reduction in VT inducibility and the overall incidence was even similar to the non-infarcted mice (34%; p<0.05) (Fig 1B). Comparable results were seen for number of VTs and duration of VT episodes (data not shown).

**Fig 2:**
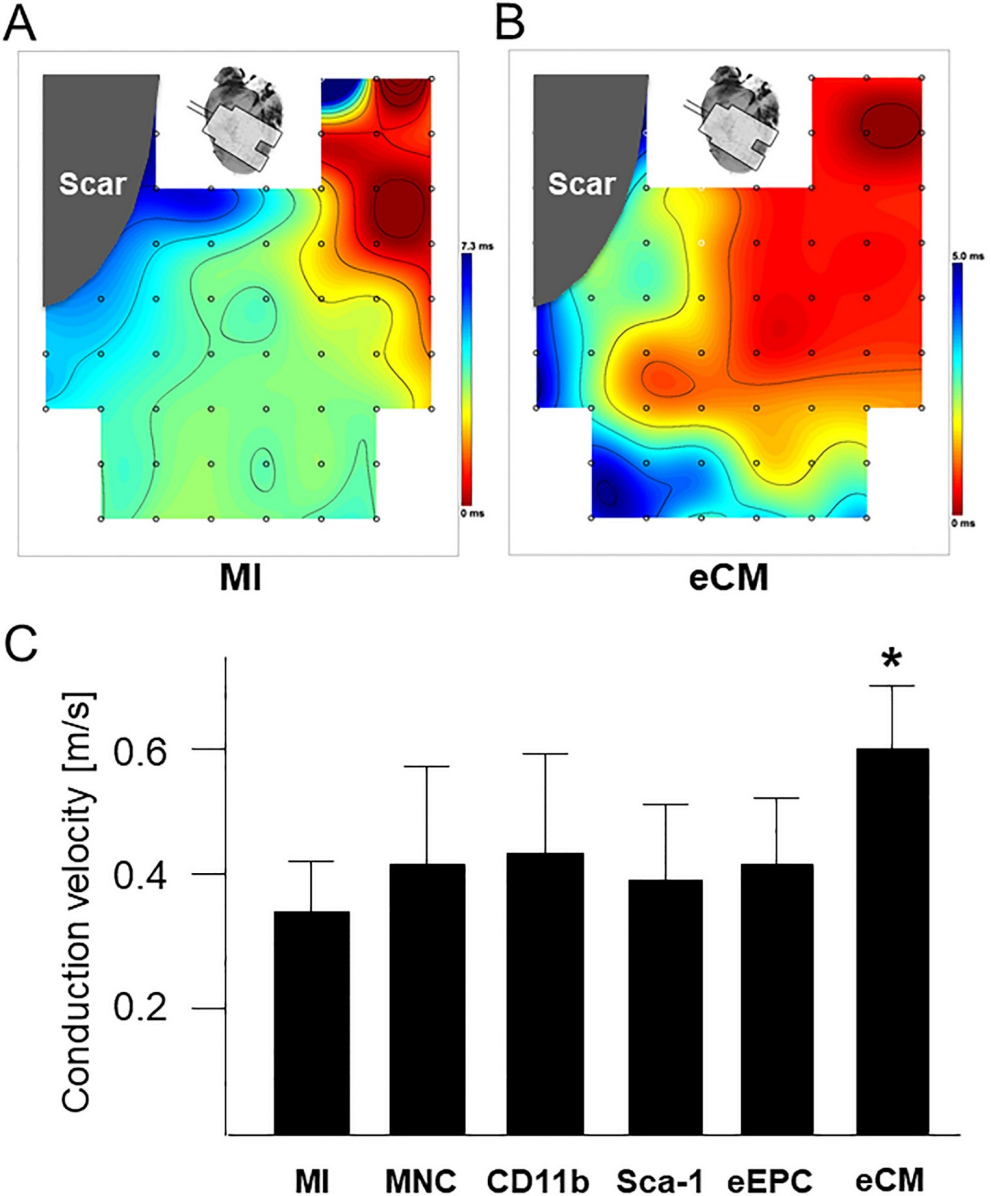
Epicardial electro-anatomical mapping of Langendorff-perfused hearts. (A and B) Representative examples of spontaneous conduction properties within the border zone two weeks after cryoinfarction in an untreated mouse (A) and after eCM transplantation (B). (C) Calculated conduction velocities in mice transplanted with different cell populations as indicated. n = 5 per group. * p<0.05 vs. MI and MNC populations.

**Fig 3:**
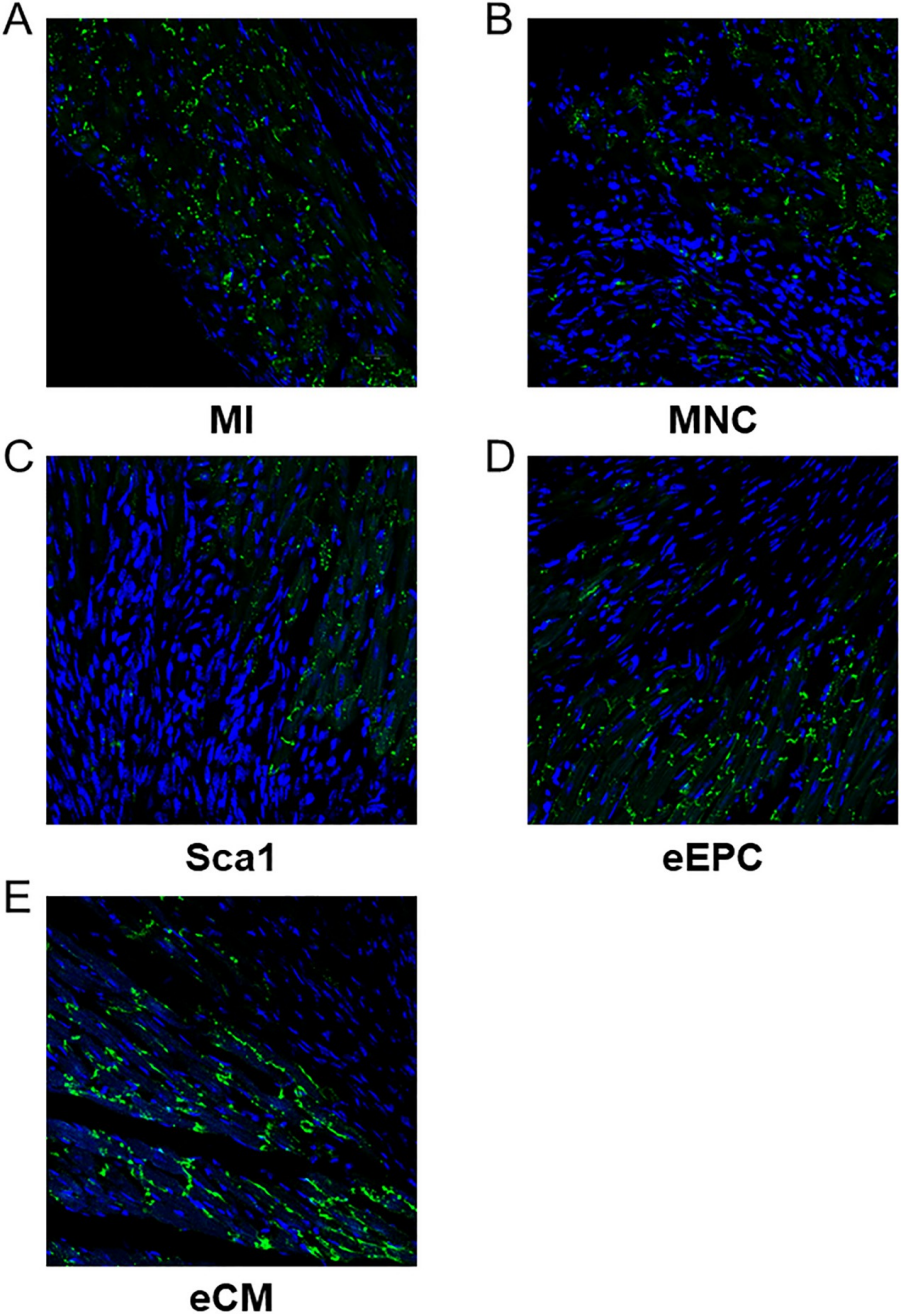
Connexin 43 expression in the border zone. (A-E) Representative immunofluorescence stainings for connexin 43 in the border zone in mice after myocardial infarction (MI) without (A) and with cell transplantation as indicated (B-E).

For analysing conduction velocities in the border zone epicardial electro-anatomical mapping in Langendorff-perfused hearts was performed. Only the eCM group showed significantly increased conduction velocities compared to the MI group (Fig 2). In accordance to these findings, Cx43 expression within the border zone appeared higher in eCM animals as compared to MNC animals (Fig 3). Of note, besides localization within intercalated discs the pattern of Cx43 expression was more punctuated in untreated or MNC treated animals (Fig 3A–3D) compared to a striped arrangement in mice receiving eCMs (Fig 3E).

### Impact of cell replacement therapy on infarct size and left ventricular function

**Fig 4:**
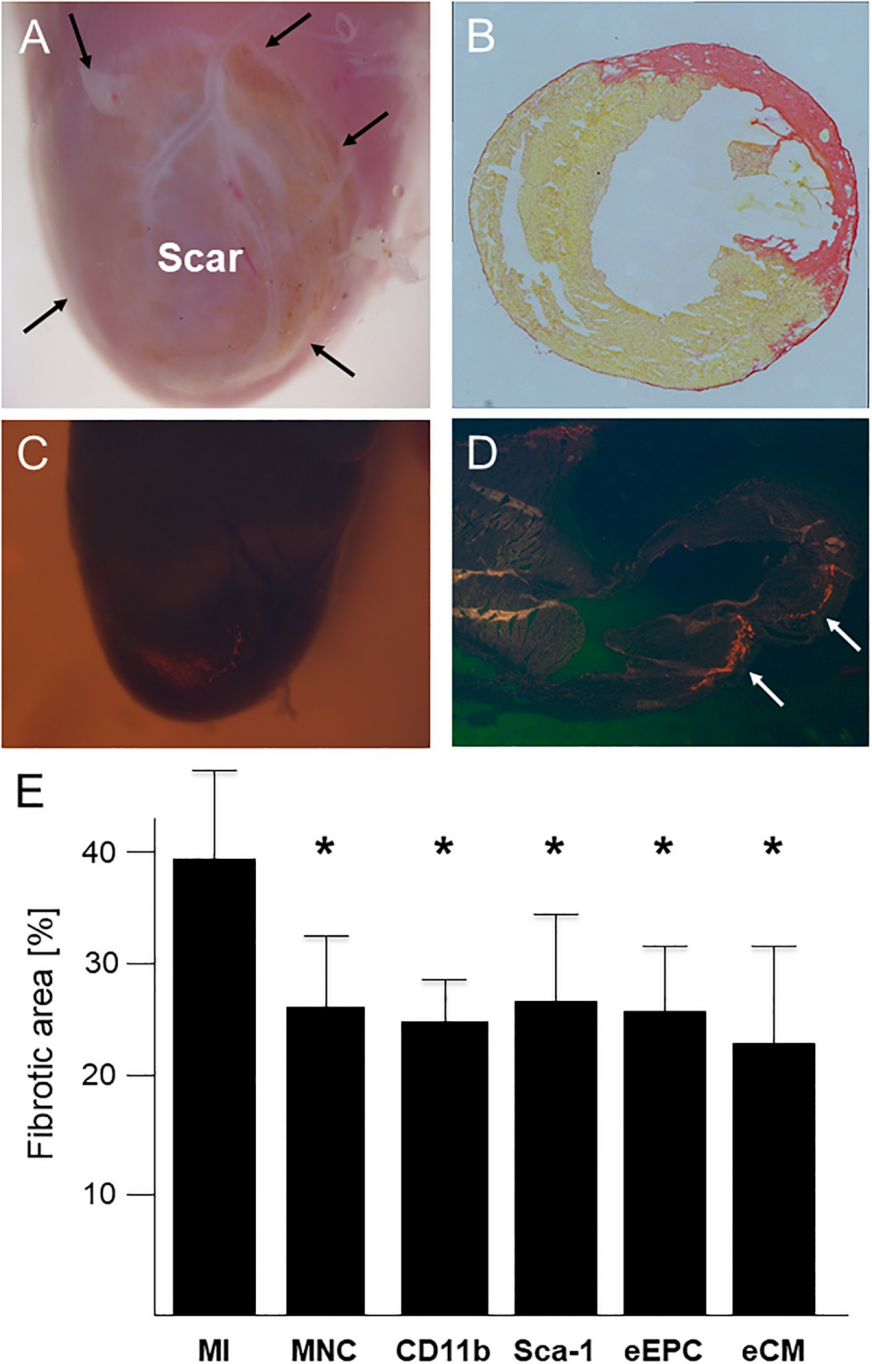
Scar formation and cell engraftment. (A) Macroscopic view of left ventricular scar with a clear-cut demarcation (arrows) to the non-infarcted tissue. (B) Representative Sirius red staining showing transmural scar formation two weeks after cryoinfarction. (C and D) Macroscopic (C) and microscopic (D) view of engrafted red fluorescent MNCs (arrows) isolated from Ds-Red.T3 mice three days after cryoinfarction. (E) Quantification of fibrotic area as percentage of left ventricle two weeks after myocardial infarction (MI) within the treatment groups. n = 5–10 per group. * p<0.05 vs. MI.

Macroscopic and microscopic analyses evidenced that cryoinjury resulted in reproducible transmural lesions (Fig 4A and 4B). Red fluorescent isolated MNCs, CD11b-positive cells, Sca-1-positive cells and eEPCs were observed at day 3 after transplantation within all cryolesioned areas displaying successful engraftment of the transplanted cells (Fig 4C and 4D). At day 14 after cell transplantation 47% of MNC transplanted hearts showed presence of fluorescent cells in microscopic analyses, 34% of CD11b transplanted hearts, 39% of Sca-1 transplanted hearts and 53% of eEPC transplanted hearts. In contrast, all eCM transplanted hearts showed engraftment of GFP-positive cells 14 days post infarction in microscopic analyses. Macroscopic analysis of infarcted hearts revealed the numerically largest relative area of engraftment following transplantation of embryonic cardiomyocytes (3.6 ± 1.1%; compared to MNC: 1.1 ± 0.5%, Sca1: 1.8 ± 0.7% and eEPC: 0.8 ± 0.2%; p = 0.0825 for overall comparison). No differences in distribution of engrafted cells could be detected between eCMs and MNCs or respective subpopulations. Engraftment was restricted to the scar area, where cells had been injected directly after the initial cryoinfarction. Sirius red staining revealed a left ventricular fibrosis area of 39.8 ± 7.6% in infarcted mice (Fig 4E). Scar tissue was significantly reduced in all treatment groups (MNC: 25.9 ± 6.1%, CD11b: 23.8 ± 3.7%, Sca-1: 26.4 ± 8.0%, eEPC: 24.2 ± 5.6%, eCM: 21.3 ± 8.4%; p<0.01 each; Fig 4E).

**Fig 5:**
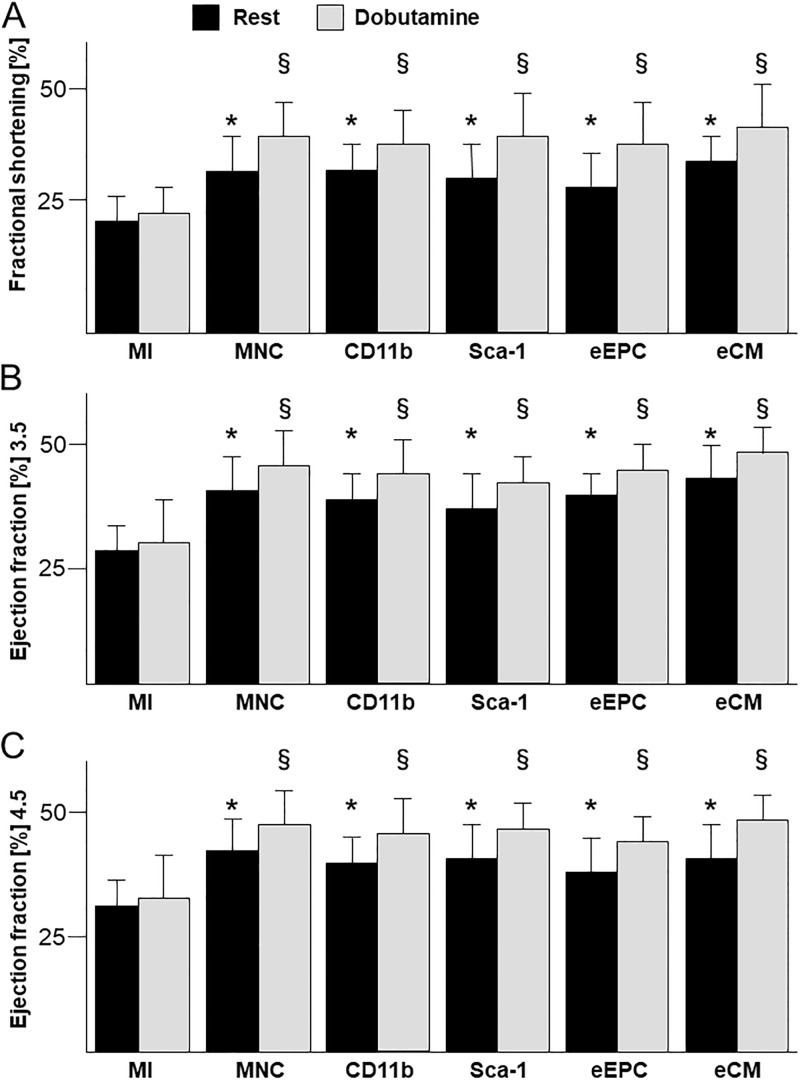
Echocardiographic assessment of left ventricular function. (A) Fractional shortening (FS) two weeks after myocardial infarction (MI) and cell transplantation at rest and after dobutamine stress. (B and C) Left ventricular ejection fraction (EF) measured 3.5 mm (B) and 4.5 mm (C) apical of the aortic valve level. n = 15–20 per group. * p<0.05 vs. MI. § p<0.05 dobutamine vs. rest.

One dimensional M-mode echocardiography as well as 2D echocardiography demonstrated impaired systolic LV function following MI at day 14 (FS: 20.2 ± 4.9%, EF 3.5: 28.3 ± 5.3%, EF 4.5: 30.5 ± 5.4%; Fig 5). Left ventricular function at rest was significantly improved in M-mode and 2D echocardiography in all treatment groups compared to untreated infarcted animals (Fig 5). In difference to untreated mice, all treatment groups revealed a significant increase of left ventricular function during inotropic stimulation with dobutamine (Fig 5), indicating preserved global inotropic response to dobutamine.

**Fig 6:**
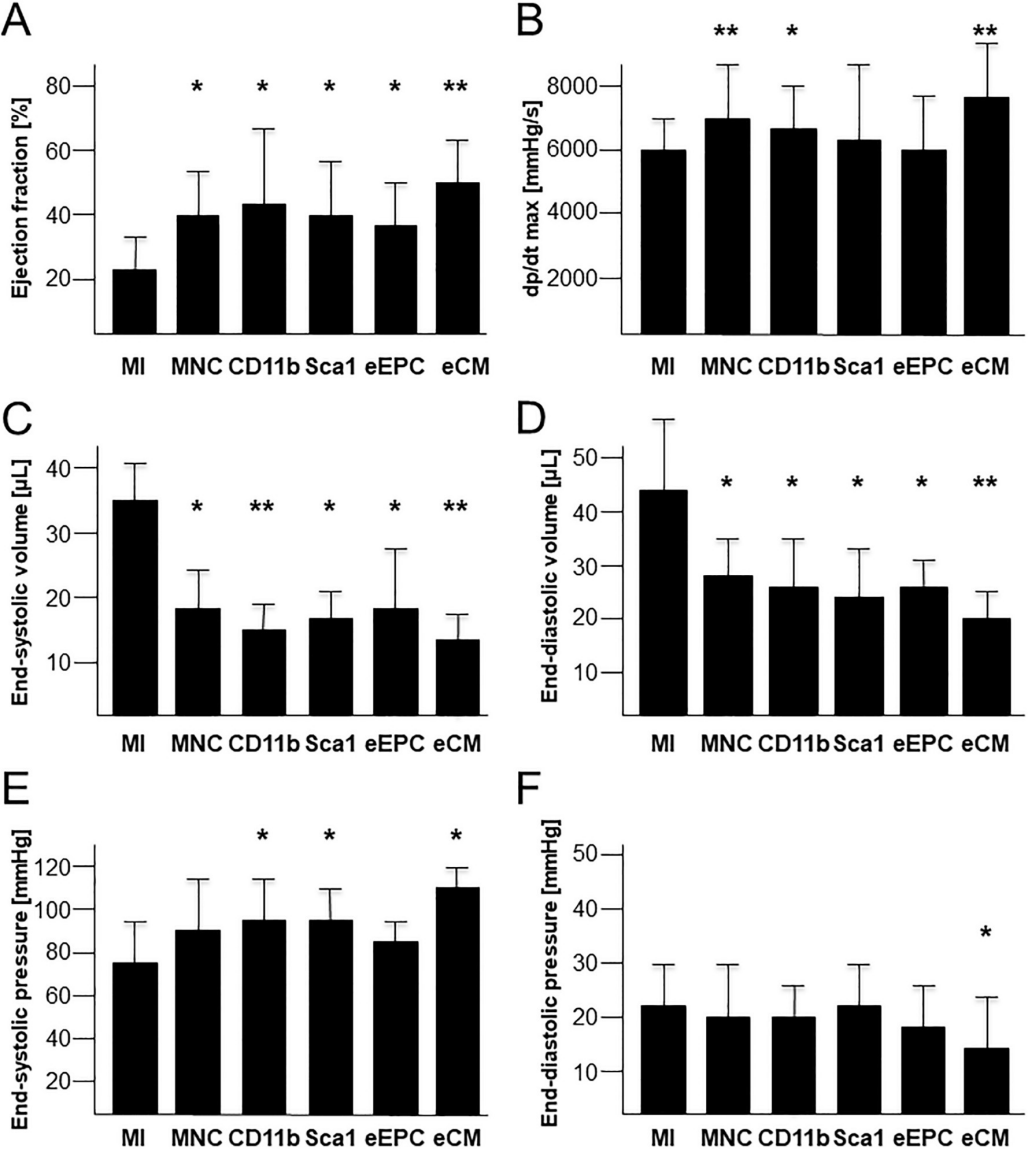
Assessment of cardiac function by left heart catheterization. (A-F) Left ventricular ejection fraction (EF; A), peak rate of rise in LV pressure (dP/dt_max_; B), end-systolic (C) and end-diastolic (D) volumes as well as end-systolic (E) and end-diastolic (F) pressures measured two weeks after myocardial infarction (MI) and cellular cardiomyoplasty as indicated. n = 15–20 per group. * p<0.05 vs. MI. ** p<0.01 vs. MI.

As further diagnostic tool left heart catheterization was performed. In accordance to data of echocardiography, EF was significantly improved in all treatment groups compared to untreated animals (Fig 6A). Contractility was significantly enhanced in eCM, MNC and CD11b treated mice (Fig 6B), characterized by an increased dP/dt max. We observed an improvement of left ventricular dilation as evidenced by a significant decrease in end-systolic and end-diastolic volumes in all treatment groups (Fig 6C–6D). End-systolic and end-diastolic pressures were reduced in CD11b/Sca1/eCM treated or only eCM treated mice, respectively (Fig 6E–6F).

### Discussion

Our data demonstrates that transplantation of different MNC populations improves left ventricular (LV) function after cryoinfarction but does not prevent post-infarction ventricular arrhythmia. In contrast, transplantation of eCMs improved both LV function and electrical vulnerability.

In the present study the cryoinfarction model was used because it is characterized by specific remodelling factors that are favourable for cellular engraftment [25]. Moreover, lesion size is reliably reproducible and lesions are transmural. Cryoinfarction induced a broad impairment of left ventricular function. Moreover, infarcted animals without cell therapy showed a high ventricular vulnerability in electrical *in vivo* stimulation as it was shown earlier by our group [10]. Therefore, this model is highly conclusive for testing therapeutic effects of cellular replacement therapy.

Main finding in our study was a reduced inducibility of VT only in the eCM group. Ventricular refractory period, an important determinant in arrhythmogenesis, was normalized within the eCM group but not within the MNC groups. In addition, epicardial mapping revealed increased conduction velocities within the border zone in the eCM group which is an important finding explaining reduced ventricular vulnerability. Congruent with these improvements of electrophysiological properties of the heart, Shiba et al. [11,12] observed a significant reduction of spontaneous and electrically stimulated VTs in cryounjured guineapigs. Yet the same researches also described a pro-arrhythmogenic potential of CMs derived from induced pluripotent stem cells in monkeys [13]. Furthermore, Liao et al. observed a higher prevalence of inducible VTs in a mouse infarction model [14], showing that the effect of cell transplantation on arrhythmogenicity is highly and controversially debated among researchers and by now not fully understood.

eCMs showed persistent engraftment after 14 days in all transplanted animals. In addition, there appeared to be an increased expression of Cx43 in this group compared to the MNC groups. An enhanced electrical coupling of cells reduces vulnerability to VT by decreasing the incidence of conduction blocks within the infarct and/or by a modulatory effect on border zone cardiomyocytes [10]. Expression of the cardiac gap junction protein Cx43 is the critical factor underlying augmented intercellular electrical conduction and protection from arrhythmia [2]. In a previous work of our group, Ca^2+^ signals from engrafted eCMs expressing a genetically encoded Ca^2+^ indicator revealed functional coupling of the graft with the native myocardium [10]. Engraftment of Cx43-expressing SMs also protected against VT induction whereas non-Cx43 expressing SMs increased the risk for VT [10]. The difference to MNCs is in fact that eCMs express functional gap junctions and can electrically couple with the native myocardium therefore potentially granting action potential propagation through scar tissue.

Transplantation of MNCs, CD11b-positive-cells, Sca-1-positive cells and eEPCs resulted in a considerable reduction of the left ventricular fibrotic area consistent with an improvement of left ventricular function similar to the effects seen in the eCM group. Therapeutic effects on left ventricular function were verified by both echocardiography and left heart catheterization. In addition, inotropic stimulation with intravenous dobutamine increased global systolic LV function in the treatment groups but not in the MI control group. Moreover, our data illustrated effects of cellular cardiomyoplasty also on diastolic function. These findings are in accordance to many preclinical studies that confirmed improved LV function after transplantation of different cell populations [26–28]. A comparative study of bone marrow MNCs, mesenchymal stem cells, SMs and fibroblasts revealed the best improvement of cardiac function in the MNC group [29]. For the MNC subgroup of EPCs, an improvement of LV function has also been shown [30,20]. Although the majority of experimental studies have presented encouraging results, clinical outcomes are divergent [3]. Differences in cell type and dose, study design, patient populations and timing of cell transplantation could explain inter-study discrepancies. A further explanation could be that preclinical studies are performed in healthy animals whereas clinical trials are performed in patients with multiple risk factors that could affect the health of BM. Recently, a meta-analysis with more than 1,600 patients demonstrated a moderate but significant improvement of LV function after treatment with autologous BMCs [5]. In particular younger patients and patients with more severely depressed LV function had the largest benefit.

In the present study, beneficial effects of MNC populations on LV function were established even though, in contrast to eGFP-positive eCMs, detection of Ds-Red-positive MNCs in the myocardium/scar tissue decreased substantially after 14 days. The majority of grafted cells has been reported to disappear within a short time period when directly injected into ischemic hearts [31]. A paracrine mechanism has been proposed as possible mediator of beneficial effects following transplantation of MNCs [32], as BM cells are known as a natural source of multiple cytokines. Cho et al. could show that intramyocardial EPC transplantation induces humoral effects that are sustained by host tissues after disappearance of transplanted cells [31]. This may play a crucial role in repairing myocardial injury by induction of higher capillary density, higher cell proliferation rates and less cardiomyocyte apoptosis resulting in reduced infarct size and better left ventricular performance [31]. Paracrine mechanisms may also be involved in anti-arrhythmic effects of transplanted BM derived cells. Kuhlmann et al. showed that G-CSF/SCF treatment partially reversed down-regulation of Cx43 expression in the border zone of infarction, resulting in reduced ventricular arrhythmia inducibility during *ex vivo* stimulation [33]. In a rat model of chronic MI, local injection of growth factor induced mobilization of cardiac progenitor cells and resulted in reduced arrhythmogenesis by prolongation of VRP and increased Cx43 expression [34]. In contrast, in our animal model, after transplantation of MNC populations, there was only a trend towards a lower incidence of ventricular arrhythmia.

### Limitations

Although, the cryoinfarction model perfectly enables the evaluation of different therapeutic approaches on ventricular arrhythmia susceptibility and favours engraftment of transplanted cells [10,24], the alternative ligation of the left anterior descending artery represents the more physiological model of MI. We only investigated the effects of cell transplantation on cardiac function and arrhythmia vulnerability 14 days after MI. It is crucial and still has to be investigated whether the demonstrated beneficial effects will be conserved over a longer period of time.

### Conclusions

Transplantation of MNCs, CD11b-positive cells, Sca-1-positive cells and eEPCs after myocardial infarction leads to an improved left ventricular function similar to effects of eCMs. In contrast to eCMs, MNC populations did not prevent post-infarction arrhythmia.

## Supporting information

S1 Dataset (SAV)
